# Discrimination of cortical laminae using MEG

**DOI:** 10.1016/j.neuroimage.2014.07.015

**Published:** 2014-11-15

**Authors:** Luzia Troebinger, José David López, Antoine Lutti, Sven Bestmann, Gareth Barnes

**Affiliations:** a Wellcome Trust Centre for Neuroimaging, Institute of Neurology, UCL, 12 Queen Square, London WC1N 3BG, UK; b Sobell Department of Motor Neuroscience and Movement Disorders, Institute of Neurology, UCL, Queen Square, London WC1N 3BG, UK; c Electronic Engineering Department, Universidad de Antioquia, UdeA, Calle 70 No. 52-21, Medellín, Colombia; d LREN, Département des Neurosciences Cliniques, CHUV, University of Lausanne, Lausanne, Switzerland

## Abstract

Typically MEG source reconstruction is used to estimate the distribution of current flow on a single anatomically derived cortical surface model. In this study we use two such models representing superficial and deep cortical laminae. We establish how well we can discriminate between these two different cortical layer models based on the same MEG data in the presence of different levels of co-registration noise, Signal-to-Noise Ratio (SNR) and cortical patch size. We demonstrate that it is possible to make a distinction between superficial and deep cortical laminae for levels of co-registration noise of less than 2 mm translation and 2° rotation at SNR > 11 dB. We also show that an incorrect estimate of cortical patch size will tend to bias layer estimates. We then use a 3D printed head-cast (Troebinger et al., 2014) to achieve comparable levels of co-registration noise, in an auditory evoked response paradigm, and show that it is possible to discriminate between these cortical layer models in real data.

## Highlights

• We evaluate necessary recording precision to distinguish superficial/deep laminae.
• For coregistration error of < 2 mm/2° we can distinguish between these laminar models.
• Incorrect assumptions about cortical patch size bias these layer estimates.
• Initial results suggest that the auditory M100 derives from deep cortical layers.

## Introduction

Magnetoencephalography (MEG) non-invasively measures the changes in magnetic fields outside the head which are caused by neuronal current flow. As MEG is a direct measure of this current flow, the achievable spatial resolution is not bounded by physiology or anatomy (for example, there are no physical limitations due to local vasculature or capillary bed structure, as in PET or fMRI). Rather, the bounds on MEG spatial resolution come from the models used to explain the MEG data. The higher the signal to noise ratio (SNR) – due to better experimental design, more sensors or longer recording sessions – the more complex the model we can propose. Consequently, modelling error becomes the main limiting factor to MEG spatial resolution. In practice, a great deal of this modelling error is due to limited knowledge of cortical anatomy with respect to sensor positions.

Head movement during MEG recordings is usually not fully constrained and co-registration to anatomy relies upon matching a small number of fiducial locations or matching two smooth round surfaces. This means that the accuracy of the information we have regarding true head position – an important factor our source and forward models rely on – is often limited. For example, using conventional co-registration techniques (i.e. fiducial markers placed on anatomical landmarks), co-registration error is typically assumed to be of the order of 5 mm (Singh et al., 1997, Stolk et al., 2013).

We recently introduced a technique which leads to dramatic reductions of co-registration error by restricting head movement through the use of 3D printed, subject-specific head casts (Troebinger et al., 2014). This gives access to very high signal to noise ratio (SNR) data sets in which the mapping onto underlying anatomy can be achieved with high precision. Furthermore, our work showed how the MEG data could be used to discriminate between an anatomical model based on the subject's cortex and an anatomical model based on another individual's cortex warped into the same space. In the present paper we sought to take this a step further and attempt to discriminate between cortical models representing the deep and superficial cortical laminae of the same individual.

In order to be able to detect changes in electromagnetic fields induced by neural activity outside the scalp, two conditions have to be met by the neuronal generators of that signal. First, that the architecture of the neuronal cell is such that it supports and gives rise to a large net current flow, and second, that neighbouring cells drive their intracellular currents with a high degree of group synchronization, so that activity adds up and produces a signal detectable at some distance. These criteria are satisfied by the large populations of pyramidal cells in layers II/III and V of the neo-cortex. Traditionally, they are assumed to form the largest contribution to the MEG signal detected at the sensors (Hamalainen, 1992, Murakami et al., 2002). However, possible contributions from other cell types have been discussed in recent years. For instance, Murakami and Okada (2006) found that, whilst layer V and II/III pyramidal cells were capable of producing very strong (0.29–0.90 pA) electrical dipoles (Q), both spiny and aspiny stellate cells, which make up the majority of layer IV neurons, produce a dipolar current of sizeable magnitude (0.27 pA and 0.06 pA, respectively). Other distinctions between cell types can be observed in their local connectivity. Layer V pyramidal neurons receive the greatest lateral trans-columnar input (Schubert et al., 2007), whereas in LII/III, the probability of a lateral connection significantly decays over a spatial scale of ∼ 150 μm (Holmgren et al., 2003). In addition to this, layer V pyramidal neurons are generally longer with thicker dendrites than those in layer III and will therefore have a greater dipole moment for the same current flow per unit volume (Jones et al., 2007, Murakami and Okada, 2006).

A number of elegant biophysical time-series models have been proposed and implemented (Bastos et al., 2012, Jones et al., 2007) to make inference on the contributions of these different cell types. For example, Jones et al. (2007) used a biophysically realistic computational model of the primary sensory cortex in order to make laminar specific predictions of the generators of the first 175 ms of the sensory evoked response.

Our aim here is to establish the conditions necessary to spatially distinguish between activity arising in different layers of the cortex non-invasively using MEG. We use purely spatial information and assume that we are dealing with neuronal populations that are oriented normal to the cortical surface. We simulate data on one of two possible surface models corresponding to the most superficial (pial) and deepest (white/grey boundary) cortical surfaces. We then attempt to reconstruct these data onto both of these surface models and examine the evidence in favour of the correct model. We then examine this discrimination under different levels of coregistration noise and signal to noise ratio. As we know that cells in different cortical layers also have distinct lateral connectivity domains (Schubert et al., 2007) we were interested in how our prior expectations of the extent of a cortical source (Cottereau et al., 2007, Grova et al., 2006, Hillebrand and Barnes, 2002, Jerbi et al., 2004) would influence our ability to distinguish between cortical layers. Having established the constraints in simulation we then go on to show an example of an auditory evoked response, recorded using a head cast, in which significant differences between layer models are evident. This is single subject data for which we had no strong prior hypothesis; we use it principally as a vehicle to describe some of the additional methodological steps that will have to be dealt with in real data.

## Methods

### MRI acquisition

MRI data was acquired using a Siemens Tim Trio 3T system (Erlangen, Germany). The subject lay in a supine position. The body-transmit coil was located inside the bore of the scanner for detection of the MRI signal. The MRI data was acquired using a 3D FLASH sequence for optimal scanning efficiency (Frahm et al., 1986). The following acquisition parameters were used: field of view: (256, 256, 208) mm along the (phase (A–P), read (H–F), partition (R–L)) directions, and image resolution: 1 mm^3^. The repetition time TR was set to 23.7 ms and the excitation flip angle was set to 20° to yield good T1-weighted contrast, standard in most anatomical applications (Helms et al., 2008). 8 echoes were acquired following each excitation and averaged offline to produce one anatomical image with optimal signal-to-noise. A high readout bandwidth was used to preserve brain morphology and no significant geometric distortions are expected in the images. Padding was used to minimize subject motion but some residual effects might remain present in the MRI images. The total acquisition time was 21 min 07 s.

### Freesurfer surface extraction

FreeSurfer (Fischl, 2012) was used to perform extraction of cortical surfaces from the anatomical MRI of the individual subject. FreeSurfer breaks this task down into several steps. First of all, intensity correction is performed to deal with any intensity variations due to magnetic field inhomogeneity. This results in a normalized intensity image. Extracerebral voxels are then removed using a ‘skull stripping’ approach. Segmentation was performed based on the geometric structure of the grey–white interface, and cutting planes are computed to separate the cortical hemispheres and disconnect the subcortical structures. A single filled volume is produced for each of the hemispheres, each of which is covered with a triangular tessellation and deformed such that an accurate, smooth representation is formed of both the white/grey matter boundary and the pial surface. For more detailed information on the algorithmic procedures, see Dale, Fischl, and Sereno (Dale et al., 1999).

The above process yields surface extractions for the pial surface (the most superficial layer of the cortex adjacent to the cerebro-spinal fluid (CSF)), and the white/grey matter boundary (the deepest cortical layer). Each of these surfaces is represented by a mesh comprising 21,401 vertices. For the remainder of this paper, we will refer to these two surfaces as deep (white/grey interface) and superficial (grey-CSF interface).

### Multiple Sparse Priors (MSP)

We used the greedy search option of the MSP algorithm (Friston et al., 2008) implemented as outlined in Lopez et al. (2012b). The MSP algorithm requires a set of covariance matrix priors corresponding to cortical patches to be defined a priori. Each of these covariance priors corresponds to a single smooth cortical patch of activity (we did not use bilateral patches as in the original MSP formulation) and is therefore determined by the location of an impulse response on the cortical surface and a local smoothness operator determining the full-width half maximum (FHWM) or spatial extent of the source. In this case we had N = 48 such priors for the simulation studies and 32 randomly chosen sets of N = 512 patches/priors for the real data (see later). We also varied the smoothness of the cortical distribution to see if under- or overestimating this parameter would tend to bias us towards deep or superficial layers.

MSP returns a Free Energy value which approximates the model evidence for the final generative model. Since this generative model includes the cortical surface model used as well as the lead fields, it can be used to compare between different models (Henson et al., 2009, Lopez et al., 2012b). In this paper we show mean log model evidence differences over simulations, i.e. the difference in log model evidence which one would expect on any single realisation (a much finer distinction being possible for the whole group of simulations).

In brief, the MEG data can be related to the neural activity that generates it using the linear model:

(1) Y=KJ+ε

where $Y\in {ℜ}^{N_{c}\times N_{t}}$ is the sensor data, where *N*_*c*_ = 274 is the number of sensors (normally 275 but one channel turned off) and *N*_*t*_ is the number of time samples; $K\in {ℜ}^{N_{c}\times N_{d}}$ is the lead field matrix that maps the *N*_*d*_ source locations to the *N*_*c*_ channels; $J\in {ℜ}^{N_{d}\times N_{t}}$ is the current distribution at each source location; and ϵ is zero mean Gaussian noise. We used a single shell (Nolte, 2003) based on the inner surface of the skull to define the forward model.

Under Gaussian assumptions, for known source covariance, **Q**, the current distribution $\hat{J}$ can be estimated directly:

(2) $$\hat{J}=QK^{T}{\left(\sum_{\varepsilon }+\mathrm{KQ}K^{T}\right)}^{-1}Y$$

where *^T^* denotes a matrix transpose. Here we assume that sensor noise **Σ**_*ϵ*_ = *h*_0_***I***_*Nc*_ is independent and uniformly distributed, with $I_{N_{c}\;}$ an (*N*_*c*_ × *N*_*c*_) identity matrix and *h*_0_ a hyperparameter effectively controlling the regularisation. Different M/EEG algorithms entail different choices of the prior source covariance ***Q*** (Friston et al., 2008, Wipf and Nagarajan, 2009). For the minimum norm (MNM) solution, ***Q*** is simply an (*N*_*d*_ × *N*_*d*_) identity matrix; for the Multiple Sparse Prior (MSP) solution, ***Q*** comprises an optimised mixture of a library of *N*_*q*_ source covariance components $C=\left\{C_{1},\ldots ,C_{N_{q}}\right\}$:

(3) $$Q=\sum_{i=1}^{N_{q}}h_{i}C_{i}.$$

Each source covariance component ***C***_***i***_ is generated as a bell-shaped smoothed region with a maximum over its centre (Harrison et al., 2007). This shape is formed with a Green's function over a graph Laplacian. The Green's function *Q*_*G*_ of (*N*_*d*_ × *N*_*d*_) is defined as:

(4) $$Q_{G}=e^{\sigma G_{L}}.$$

With *σ* a parameter that defines the size of the bell, and *G*_*L*_ of (*N*_*d*_ × *N*_*d*_) the graph Laplacian generated with the vertices and faces provided by the cortical surface:

$$G_{L}=\left\{\begin{array}{l} -\sum_{k=1}^{N_{d}}A_{\mathrm{ik}}\;,\mathrm{for}\;i=j,\;\mathrm{with}\;A_{i}\;\mathrm{the}\;i‐\mathrm{th}\;\mathrm{row}\;\mathrm{of}\;A\\ \;A_{\mathrm{ij}}\;,\;\mathrm{for}\;i\neq j \end{array}\right)\;$$

where *A* is an adjacency matrix of (*N*_*d*_ × *N*_*d*_), with *A*_*ij*_ = 1 if there is face connectivity between vertices *i* and *j* (maximum six neighbours for each vertex). For a more detailed discussion of these patches and their implementation in SPM see (Belardinelli et al., 2012, Lopez et al., 2014).

The algorithm then uses a non-linear search to optimise the hyperparameters using the variational free energy as a cost function (Friston et al., 2008). Briefly, the negative variational free energy is a trade-off between the accuracy of the model in explaining the data, and the complexity of achieving that accuracy (Penny et al., 2010).

In this paper optimization is performed at two levels: within layer and across layers. Initially, each **C**_*i*_ corresponds to single local smooth patch of cortex. We used two possible levels of smoothing corresponding to FWHM = 5 and 10 mm over the cortex. In this case, where there are many hyperparameters, the optimization is achieved using a Greedy Search algorithm (Friston et al., 2008). This optimisation is carried out independently (on the same data) for each cortical layer model and returns lower bound on the free energy or an approximation to the evidence for that surface model. The selection of the centres of these patches is a problem in practice, as there is a trade off between having a large number of patches entailing a large computational and optimization burden; and a small number of patches meaning that the cortex will be subsampled. For simulations we used the same prior and simulated source locations so this was not an issue. For the empirical data however we took 32 sets of 512 randomly selected vertex indices to comprise the prior set (Lopez et al., 2012a) and used the prior with the highest model evidence.

In the empirical section, we wanted to pool the source estimates from across two independent layer models (each with *N*_*d*_ vertices) into a single two layer model (with 2*N_d_* vertices). In this case each **C**_*i*_ became (2*N*_*d*_ × 2*N*_*d*_) diagonal matrix with either the upper or lower *N*_*d*_ elements set to the source covariance estimate from one of the two (individually MSP optimised) source covariance matrices ***Q*** from Eq. (3). After optimization this gives a new (2*N*_*d*_ × 2*N*_*d*_) source covariance estimate **Q**_*both*_ which can be substituted into Eq. (2) to produce a cortical current estimate distributed over two cortical layers.

### Simulations

**Fig. 1:**
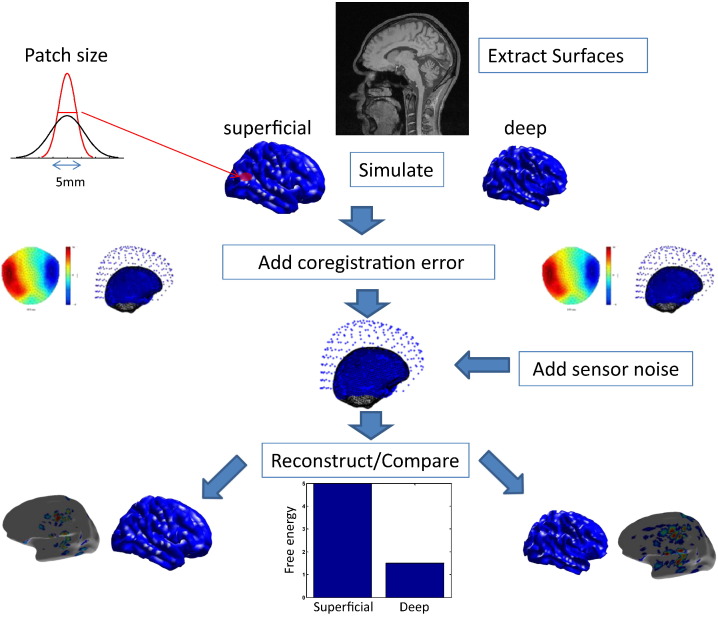
Outline of the simulation process. We use FreeSurfer to extract the pial and white matter surfaces for a single male subject. The pial and the white matter surfaces define the superficial and deep generative surface models. Activity is simulated by randomly selecting source locations from a list of 21401 possible patch centres. Data are generated using two different patch sizes; corresponding to either FWHM = 5 mm or FWHM = 10 mm (top left inset panel). Sources are simulated on both surfaces, followed by co-registration of each dataset to both cortical models. To add coregistration error, a random perturbation is added to the fiducial locations at this stage, either taking the form of rotation or pure translation of zero mean and specified standard deviation (0, 1, 2, 5, 10, 20 mm/degrees). Finally, we use the MSP algorithm to perform source reconstruction. This yields a free energy value as an outcome, which approximates the log model evidence, allowing us to compare between reconstructions based on the ‘correct’/‘incorrect’ surface models, as well as ‘correct’/‘incorrect’ patch sizes.

The basic experimental procedure is outlined in Fig. 1. On any single iteration, the first step is to generate a synthetic data set. All simulations were based on a single dataset acquired from a real experimental recording using a CTF 275 channel Omega system (Troebinger et al., 2014). The sampling rate was 600 Hz and the dataset consisted of 145 trials each with 1201 samples. We used a single shell forward model (Nolte, 2003).

At each iteration, we drew three vertex indices from a total of 21,401 indices to specify simulated source centres. These same indices were used to index cortical locations on both superficial and deep laminae (in Freesurfer the vertices remain ordered such that the vertex *n* on the pial surface is adjacent to vertex *n* on the white matter surface). We simulated sinusoidal activity profiles of 20, 22, and 24 Hz for the three sources over a time window from − 100 to 300 ms. This meant that for each iteration, we had two datasets: one corresponding to data generated by sources on either superficial or deep cortical layers, sitting at the same approximate location (i.e. differing by the cortical thickness).

We repeated this process for 16 iterations giving 32 data sets post-simulation (16 source triplets simulated on the superficial surface, and 16 triplets on the deep surface). Gaussian white Noise was then added to the simulated data to give per-trial amplitude SNR of 0 (default), − 10 or − 20 dB. Co-registration noise was added by either translating or rotating the fiducial locations by a random amount drawn from a distribution of zero mean and specified standard deviation (20, 10, 5, 2, 1, and 0 mm and 20, 10, 5, 2, 1, and 0° respectively).

We then reconstructed these data (with both sensor and co-registration noise) onto both surface models using the MSP algorithm (above). For each surface model we recorded the negative free energy.

**Fig. 2:**
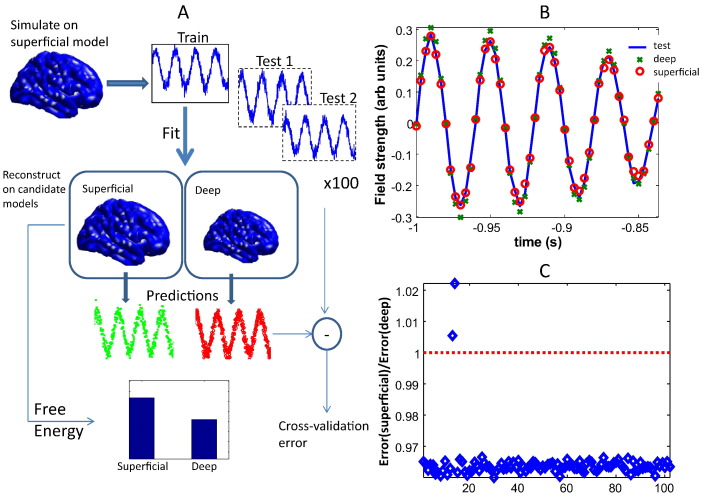
An example to show the link between the free energy metric used in this paper and traditional cross-validation approaches. The basic approach we used is illustrated in Panel A. Here, we simulate 3 sources on the deep surface, and reconstruct these data onto two candidate cortical models (superficial and deep). In this case the free energies (or log model evidences) for the two models differ by 4.3. This suggests that the pial candidate model is more likely with a probability of 1/(1 + exp(− 4.3)) = 0.9866. An alternative method to judge between models would be to use cross-validation to see which model predicts new data more accurately. Based on the original set up we simulated a further 101 data sets, using the same source locations on the superficial surface (in other words these data had exactly the same underlying signal but different noise realisations). We now use the two candidate models to generate data and compare these predictions with new test data. Panel B shows the signal for a single MEG channel, for a single test dataset (blue solid) and predictions from the two candidate reconstructions (deep model green crosses, superficial model red circles). Note that the error between the superficial candidate model and the test model (based on superficial) is smaller than that of the deep. Panel C shows the ratio of these errors (over all channels and time points) for the two candidate models over 101 test datasets. The red line is at unity, points above the line show smaller error for the candidate deep surface model, points below indicate that the superficial model provides a better prediction of the test data. The incorrect model (deep surface model) is favoured in only two cases. This means that the deep model is more likely with a probability of 2/101 = 0.9802, in accord with the analytically derived posterior probability based on free energy (0.9866).

In order to give some insight into the free energy metric of model fit used in this paper we show the parallels with cross-validation accuracy in Fig. 2. Cross validation involves the partition of the data to be fit into training and test portions. The idea is to fit models to the training data and then to judge between models based on the test data. Models which are too complex will overfit the training data (i.e. fitting the noise) and therefore perform poorly on the test data. Similarly models which are too simple will also not fit the test data. In other words there is the same accuracy-complexity trade off as with free energy. Much like arguments for parametric and non-parametric statistics, the free energy approximation will be more powerful (as it uses all the data) when the underlying assumptions are met, whereas cross validation will be not quite as sensitive, more time consuming, yet robust. Here (Fig. 2A) we generate 102 sets of data based on a triplet of sources sitting on the superficial surface model. All these datasets have the same underlying signal but differ in their noise content. We then fit just the first (training) dataset using superficial and deep candidate surface models. Each of these fits returns a free energy value and the difference between them gives us an analytic estimate of the posterior probability of one model over the other. In this case the log free energy difference between models is 4.3, meaning that the superficial candidate is the most likely with posterior probability of 0.9866 (1/(1 + exp(− 4.3))). We can now pursue an alternative route to evaluate the two candidate models by comparing the prediction of the data made by each of the models with the remaining 101 test datasets. In Fig. 2B one channel from one test data set (blue solid) is plotted alongside predictions from the superficial (red circles) and deep (green crosses) candidate models (which were fit to the training data). Note that the superficial model makes the more accurate prediction of the new test data. Now we can compare the errors (over all channels and time points) for each test dataset from the two models. Fig. 2C shows the ratio of these errors for the 101 test datasets. In 99 of these cases the error for the superficial model is smaller than that of the deep model (points below unity) — i.e., a cross validation accuracy (or non-parametric posterior model probability) of 0.9802.

### Experimental evaluation

#### Subject task

We collected data from a single male subject wearing a head cast. We used a simple finger movement task adapted from Muthukumaraswamy (2011), which involved abductions of the right hand index finger performed to an auditory cue. The cue consisted of a simple auditory tone (1000 Hz), played via a piezoelectric device connected via plastic tubing to ear-inserts, followed by an inter-stimulus interval of 3.4–4.5 s. This gave approximately 145 epochs of data per ten minute recording session. EMG traces of the first dorsal interosseus (FDI) were used to track finger movements (although we did not make use of this information directly for the purpose of this paper). Each session of scanning was split into 4 ten minute sections, during which the subject performed the finger movement task described above. Two such recording sessions were performed on separate days, giving 8 runs of task performance in total.

We used a new version of the head cast technique described in Troebinger et al. (2014). Here, rather than building a solid nylon cast we used a 3D printer (Zprinter 510) to construct a positive image of the subject's scalp surface including fiducial markers. We then used this positive image and a replica internal dewar surface to construct a polyurethane foam head-cast. Because the fiducial markers were printed onto the subject's positive head surface used to make the casts, these new head casts included designated locations for placement of fiducial coils. The standard deviation of the fiducial coil locations over the eight scanning runs was 1.1, 0.4, 0.9 mm (Nasion, nas), 0.5, 0.1, 0.3 mm (left pre-auricular point, lpa) and 0.4, 0.2, 0.6 mm (right pre-auricular point, rpa), in x, y and z, respectively.

## Results

### Simulations

#### Co-registration error

In practice, coregistration error will typically occur as a result of a combined lateral shift and rotation of the subject's model anatomy with respect to the true anatomy. Here, we simulated both of these types of error separately.

**Fig. 3:**
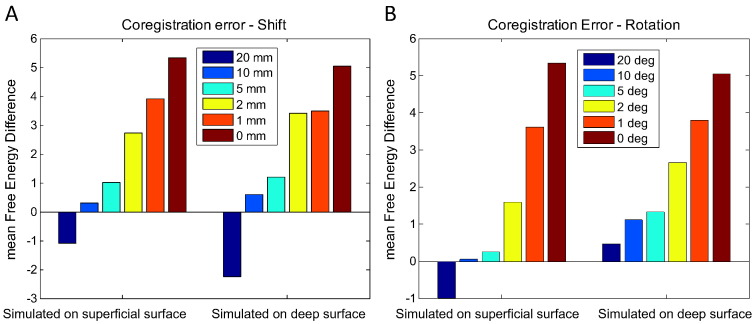
A: The effect of lateral coregistration error (shift). The bars shows the average (over triplet simulations) free energy difference between true and incorrect surface models (evidence in favour of the true model is positive) for source triplets simulated on the superficial (left) and deep (right) surface models. Different coloured bars show different levels of co-registration error in mm. Both plots indicate that the ability to discriminate between models representing different cortical surfaces is destroyed once coregistration error exceeds 2 mm (free energy differences < 3). B: The effect of rotational coregistration error. The bars shows the average (over triplet simulations) free energy difference between true and incorrect surface models (evidence in favour of the true model is positive) for source triplets simulated on the superficial (left) and deep (right) surface models. Both for simulations based on the superficial as well as the deep surface model, the cut-off for being able to distinguish between the true/incorrect surface models (with 95% certainty or free energy > 3.0) lies around the 2-degree-mark.

Fig. 3A shows the mean Free Energy difference between the reconstruction onto the true surface (e.g., simulated on the superficial layer and reconstructed on the superficial layer) and the reconstruction on the incorrect (e.g., simulated on superficial layer and reconstructed on the deep layer) surface for different amounts of coregistration error (lateral shift). The left and right sections of the plot correspond to the true models being superficial and deep surfaces respectively; evidence in favour of the true model is positive. Firstly, note that the picture from both sets of simulations is very similar (there is little asymmetry). The smaller the coregistration error the greater the model evidence difference between surface models. Mean free energy differences of greater than 3 mean that the true surface model is (on average) twenty times more likely than the incorrect one. If we take this as our significance level then note that discrimination between surfaces is only possible when coregistration error is less than 2 mm. At typical coregistration errors (of sigma = 5–10 mm) there is only marginal evidence for the true model whereas at coregistration errors of standard deviation of 20 mm, there is slightly more evidence in favour of the incorrect model. It is also interesting to note the steep rise in mean Free Energy difference moving from values of 5 mm to 1 mm of co-registration error, indicating the large amount of spatial information carried by the MEG data. It also suggests that considerable improvement lies just beyond typical (5 mm) coregistration error.

In Fig. 3B we show the effect of random rotations (rather than translations) of the MEG coordinate frame. An orientation error of 2° (with 0 translation error) would correspond, for example, to 1.4 mm lateral shift in the nasion coil with corresponding 1.4 mm shifts in the opposite direction of the left and right pre-auriculars (assuming an 8 cm head radius). For both simulations based on the superficial and deep surfaces, we can only confidently (p < 0.05) discriminate between surfaces for rotation errors of 2° or less. Although we see very little difference in the relative change of free energy for the two different surface models it does seem that the data simulated on the deeper cortical layers are more sensitive to this parameter, presumably as this deeper surface is more tightly convoluted and will produce magnetic field signatures of higher spatial frequency content.

#### Signal-to-Noise Ratio (SNR)

**Fig. 4:**
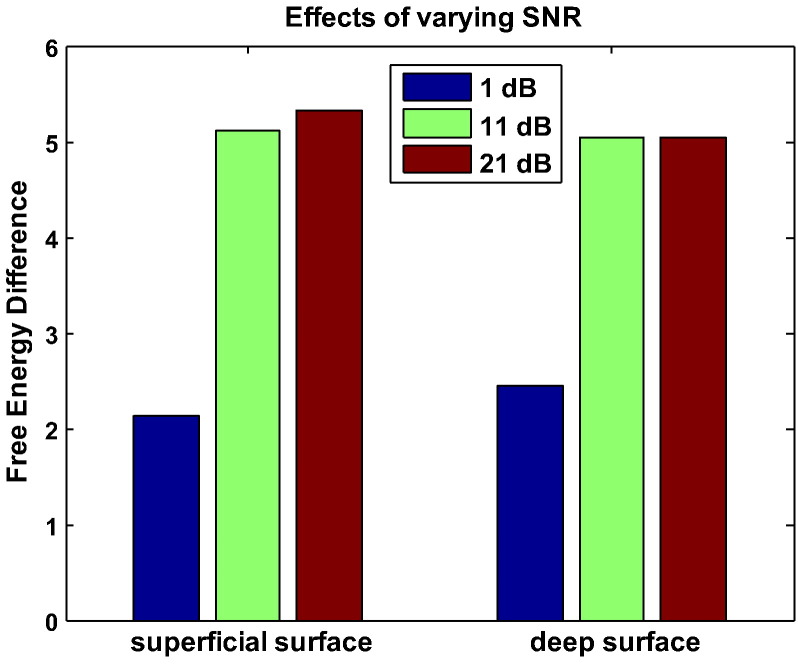
The effect of varying SNR. The ability to distinguish between surface models improves with SNR. At 1 dB SNR, it is not possible to make a significant distinction between cortical models (free energy < 3). However, at a higher, yet still moderate SNR of 11 dB, there is strong evidence in favour of the correct surface model.

Typically, MEG recorded data exhibit relatively low SNR (~ 0 dB or equivalent signal and noise r.m.s. amplitude in any single trial of data), limiting the spatial resolution at the source reconstruction stage. We wanted to test how critical a factor sensor level SNR was in the selection of the true cortical model. We simulated data at − 20, − 10 and 0 dB per trial amplitude SNR with a typical number of 145 trials. We looked at averaged data and this boosted our SNR by 21 dB to 1, 11 and 21 dB. Fig. 4 shows the average model evidence difference for reconstruction of 3 sources with zero co-registration noise. It is clear that even at 11 dB average SNR (i.e. with per trial signal amplitude at − 10 dB or 1/10th of the r.m.s. noise) it was still possible to make a clear distinction between cortical models. Whilst this may seem surprising at first glance, it is important to note that there was no coregistration noise in this case and as the simulated data were temporally smooth, the temporal dimension reduction in MSP would have also boosted the signal relative to the noise.

#### Patch extent

The MSP algorithm depends on a prior library of candidate cortical patches formed from the smoothing of a cortical impulse response. The default smoothing translates to an FWHM (or effective patch diameter) of 10 mm. We simulated sources of both FHWM = 10 and FHWM = 5 mm and then attempted to identify the true cortical surface (i.e., the surface sources were simulated on) either assuming FWHM = 10 or 5 mm. In addition to varying patch size and cortical layer we randomly perturbed rotational coregistration by sigma = 1, 2 and 5° (as in Fig. 3B). All other parameters were set to default: 0 dB per trial SNR, 3 sources, 16 iterations etc.

**Fig. 5:**
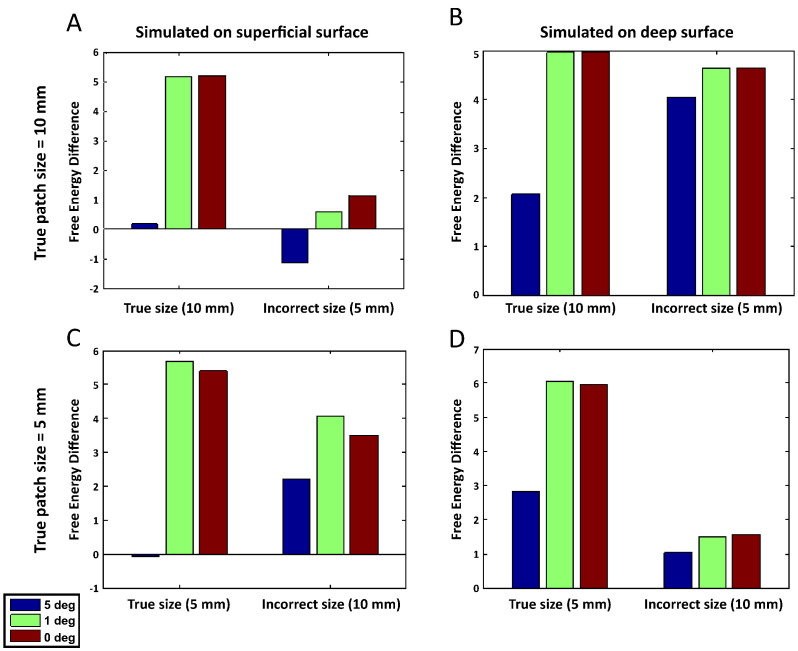
Investigating the effect of patch extent. Data were simulated on the superficial (left) or deep (right) surfaces models using either a patch extent of FWHM = 10 mm (upper panels), or FWHM = 5 mm (lower panels). Three sources were modelled, at a per trial SNR of 0 dB. In addition, rotational coregistration errors of 0, 2 and 5° were simulated. Panel A in the top row shows relative free energy differences for simulations based on the superficial surface and using a patch extent of FWHM = 10 mm. Looking at the leftmost set of bars (corresponding to reconstructions using FWHM 10 mm), we observe strong evidence in favour of the correct model (positive values) for reconstructions at true patch extent, both for 0 and 1, but not 5° of coregistration error. This pattern is destroyed when underestimating patch extent (FWHM = 5 mm), as illustrated by the rightmost set of bars where no clear difference between surfaces emerges. However, looking at Panel B, which shows the comparison based on the same patch size (10 mm), but using the deep surface model, it is clear that even if we underestimate patch extent (5 mm), the strong evidence in favour of the true model is preserved. This suggests that when we underestimate patch extent, we are introducing a bias towards deeper surface models. The bottom row shows relative free energy differences for simulations based on smaller patch extent (FWHM = 5 mm). Panels C and D correspond to the true surface models being superficial and deep, respectively. Here, in the case of superficial-surface-based data, when overestimating patch size, the evidence in favour of the superficial surface model is preserved (Panel C). On the other hand, as shown in Panel D, in the case of deep-surface-based simulations, overestimating patch size decreases the evidence in favour of the deep surface model. This indicates that by overestimating patch extent, we are introducing a bias towards superficial surface models.

The top row of Fig. 5 shows the relative model evidence differences between true (A superficial, B deep) and incorrect cortical models for simulated sources of extent 10 mm when the MSP has the correct (FWHM = 10 mm) and an underestimate (FWHM = 5 mm) of patch extent. Looking to the right of Fig. 5A we can see that if our extent prior is smaller than that simulated there is no strong evidence (free energy difference is less than 3.0) to support even superficial models with no coregistration error over deep ones. Conversely panel B right column, in which the data are simulated on the deeper surface, show very strong evidence for this surface even at coregistration errors of five degrees. Taking panels A and B together, underestimating the patch extent (FHWM = 5 rather than 10 mm) has biased the evidence in favour of the deeper cortical model. Now looking at panels C and D in which the true model is the 5 mm patch diameter, we see that there is similar discrimination between surfaces using the correct patch size priors (left most columns) as we observed for the 10 mm patches. In this case however, there is relatively strong evidence in favour of the superficial model when patch size is over-estimated (panel C, FWHM = 10 mm) but no strong evidence in favour of the deeper model even when it is the true model (panel D, FHWM-10 mm). In other words overestimation of patch extent biases the evidence towards superficial cortical layer models.

### Experimental data

**Fig. 6:**
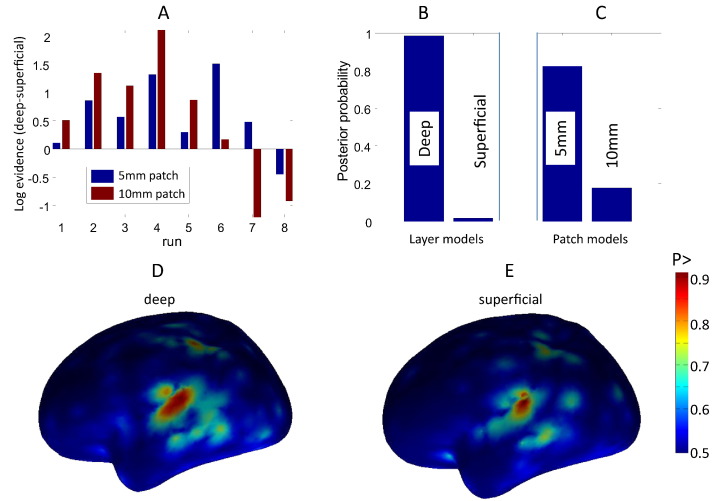
Investigation of layer and patch size models for an auditory evoked response paradigm in one subject over eight recording sessions. Panel A shows the difference in log model evidence between deep and superficial layer models for each of the eight runs for 5 (blue bars) and 10 mm (red bars) patch sizes. Positive values mean that the deep surface model is more likely; this is the case in six of the eight runs for both patch sizes. Panel B shows the probability of the two layer models based on all eight runs and both patch sizes. Note that the deeper cortical surface is more likely with a posterior probability of 0.9834. Panel C shows the probability of the different patch size models this time marginalising over layer models, the 5 mm patch size being more likely with a posterior probability of 0.8238. Panels D and E show the average posterior probability (probability that the current at any point is non zero) map for t = 0.92 s post stimulus over the eight sessions (combining both patch sizes) for deep and superficial layer models respectively.

The task we selected consisted of a button press to an auditory cue as described in Muthukumaraswamy (2010). We recorded 8 ten minute runs (of approximately 145 trials each) from a single male subject, spread over two sessions conducted on separate days. We used averaged evoked responses from 0–300 ms (0–80 Hz) time-locked to the auditory cue, baseline corrected based on − 200 to 0 ms. These data were projected into 274 orthogonal spatial (lead field) modes and 8 temporal modes. We used two different cortical layer models (deep and superficial), each with two possible patch extents (FWHM = 5 and 10 mm), to model the average auditory evoked response 0–300 ms post-stimulation in the 0–80 Hz band. For each of the 8 runs, we performed 32 MSP iterations with 512 random patch locations, using each of the two layer models, with two possible patch smoothness values. We took the best model (in terms of free energy) for each of these 4 parameter combinations. In Fig. 6 we show the difference in free energy between the best deep and superficial models for 5 mm and 10 mm patch extent. Note that the deep layer model tends to improve upon the superficial and that, although this difference is influenced by patch size, the deep layer model wins in 6 of the 8 runs. Taking account of all these free energy differences in a fixed effects manner (Penny et al., 2010, Stephan et al., 2009) allows us to compute the posterior probability of the two surface models marginalising over patch smoothness (panel B), or of the two patch smoothness options marginalising over layers (panel C). We find that the most likely origin of these data over the group of recordings is the deeper cortical layer with a posterior probability of 0.9834. The patch comparison is more equivocal with the smaller patch size having slightly greater posterior probability at 0.8238.

For each run we then pooled the source covariance estimates for each of the four best source models (deep and FWHM 5 mm, superficial and FWHM 5 mm, deep and FWHM 10 mm, superficial and FWHM 10 mm) to create a single current distribution extending over both cortical layers. In panels D and B we show the average of the eight posterior probability maps (the probability of non-zero current flow) at 92 ms post stimulus (the peak in global field power over the eight runs). Note the different current distributions on the two cortical layers; note also that although at the group level the evidence in favour of the deep layer model was overwhelming; at the individual run level the marginal (< 3) differences in model evidence between the two layer models means that the posterior current density will straddle both surfaces.

## Discussion

In this paper, we have shown that for low values of coregistration error (below 2 mm and two deg) and moderate SNR (11 dB for average data), it is theoretically possible to distinguish superficial and deep cortical laminae using MEG. In addition to exploring this in a theoretical context using simulations, we have also performed a first demonstration that differences between layer models can be observed in real MEG data, recorded using a head cast.

These results provide evidence that, with only slight modifications to current scanning paradigms and procedures, non-invasive human laminar electrophysiology is within reach. In this study we have concentrated on the distinction between the upper and lower surfaces of the cortical sheet but it is not inconceivable that finer distinctions between individual cortical laminae will be possible given the SNR levels (built up over multiple sessions) and low co-registration errors that can now be achieved using individual head casts. These laminar models could be defined through interpolation between the surface models used here or based on new in-vivo MRI techniques such as in-vivo histology (Dick et al., 2012).

One important finding was that the distinction between laminae will be biased by assumptions made about source extent: in our simulations, we showed that an underestimation of the true patch extent will tend to bias estimates towards deeper layers, whereas an overestimation of patch extent will tend to bias layer estimates more superficially. Given the pre-existing differences in lateral connectivity between cell populations in different layers (Schubert et al., 2007) this will be an important factor to marginalise out of the inference on layer specificity in future work. Also, in the same way that removal of co-registration error improves the forward model, one would expect improved layer distinction with more comprehensive volume conductor models. For example, recent work (Stenroos et al., 2014) showed that a three-shell boundary element model outperformed the single-shell model used here.

Here we used a head-cast both to remove head movements during scanning and to provide a precise mapping between the structural and functional data. If this precise mapping (between MEG fiducial coils and anatomy) was known for a subject, and the head-movements compensated for using techniques such as Signal Space Separation (SSS) (Taulu and Simola, 2006), then in principle the same layer distinctions should be possible. The only potential issue is that the distinction between laminae may depend on the number of orthogonal spatial modes in the data (which are inevitably diminished in software based approaches to head movement correction).

The finding that discrimination between layers is not particularly sensitive to SNR is encouraging. Importantly SNR here is not the signal change between conditions, or from one cortical source with respect to all others, but rather all cortical sources with respect to the system (plus environmental) noise. Here we assume Gaussian white noise for typical MEG recordings above 1 Hz; but other noise models (e.g. pink) might be more appropriate in situations where one is trying to link layer models to specific frequency bands (see later).

The empirical data raised a number of methodological issues. Classical statistics based on random field theory for multiple lamina models will become an issue here as the surfaces are very highly correlated (in MEG space), although not connected (in our anatomical model), hence random field theory (on a surface) will overestimate the intrinsic volume (or become more conservative) by at least a factor of two (for two surfaces). There could be a number of ways around this — the use of non-parametric approaches (Nichols and Holmes, 2002), the use of heuristic estimates of the effective number of independent elements (Barnes et al., 2011), or, alternatively, the warping of the set of surfaces into a common space defined by their lead fields. At the moment we are uncertain how to optimally combine data from multiple runs. Here we have opted to plot the average posterior probability map. This means pooling data from the different models (superficial, deep, 5 and 10 mm patch size) at a per-run level. Consequently the balance of current flow between the two layers is also determined by the relative model evidences for the different surface models on each individual run. One alternative approach would be to take the winning model from the group (the deep layer model in this case) and present all data from all runs on this single surface. Related to this issue, we currently perform the MSP prior optimization for each layer model independently. This allows us to make categorical statements about the most likely layer model. One could imagine a third model that would support prior current distributions over both cortical layers (or even multiple layer models). This scenario, which would allow some cortical areas to consist of predominantly deeper layer sources and others more superficial, would certainly be more realistic (especially given that typically, we are interested in time windows of hundreds of milliseconds), although potentially more computationally demanding.

We had no strong hypotheses about the layer specificity of the auditory evoked response. In this single subject, the preference for the deep layer model over the superficial layer model is clear, but we note that the invasive literature here is equivocal. Whilst some invasive studies in other species report similar magnitude responses across layers (Ogawa et al., 2011, Steinschneider et al., 2008) other studies do show a deep layer bias (Harris et al., 2011, Profant et al., 2013, Sakata and Harris, 2009). Profant et al. (2013) studied the multi-unit response properties of the auditory cortex in the rat. They found the weakest response in superficial layers, which they attribute to the lowest direct thalamic input (as well as possible influences from their recording procedure). Perhaps the strongest arguments come from the general physiology and anatomy. Investigating the laminar organization of the auditory cortex and its response to stimuli (tones, clicks) using single cell recordings, Sakata and Harris found that while layer II/III pyramidal cells exhibit selective responses in both spectral and temporal domains, layer V thick pyramidal cells do not share this selectivity, and exhibit a much more general response pattern. This specificity of superficial and generality of deeper neuronal population is linked by these authors to their distinct lateral connectivity profiles (narrow and broad respectively). Indeed we think the most plausible explanation of the preference for the deep layer model we found here is that the increased trans-columnar connectivity in the deeper layers will result in a larger synchronous population and hence a larger dipole moment. Coupled with this, layer V pyramidal neurons are generally longer with thicker dendrites than those in layer III and will therefore have a greater dipole moment for the same current flow per unit volume (Jones et al., 2007, Murakami and Okada, 2006).

In these data we also find a preference for smaller patch sizes. This parameter needs further investigation. Reliable estimates of patch size would allow us to compare directly with anatomical connectivity estimates (which may also help distinguish between layers); provide a non-invasive estimate of cortical current density; and generally help inform the basis set for other algorithms which tend to be either based around point-like (e.g. beamformers) or intrinsically smooth (e.g. LORETA) solutions.

Both empirical and modelling works show that the MEG signal is a result of multiple cell populations in different layers. Identifying a paradigm to conclusively demonstrate this layer specificity empirically will be the next hurdle. See for example recent work (Ronnqvist et al., 2013) comparing human MEG measurements with laminar in-vitro recordings.

One promising avenue is a focus on beta and gamma oscillations, for which both empirical and theoretical works suggest a predominance in deep and superficial layers respectively (Bastos et al., 2012).

This is only the first step towards empirical validation and requires replication using empirical data from several subjects and different paradigms. We should also note that here we have used purely spatial models and made no use of the time-series information (Bastos et al., 2012, Jones et al., 2007, Moran et al., 2008). Future work might consider the comparison and eventual combination of such techniques. To conclude, here we have provided first evidence that non-invasive human laminar electrophysiology is practically achievable.
